# A Mass Spectrometry-Based Assay for Improved Quantitative Measurements of Efflux Pump Inhibition

**DOI:** 10.1371/journal.pone.0124814

**Published:** 2015-05-11

**Authors:** Adam R. Brown, Keivan A. Ettefagh, Daniel Todd, Patrick S. Cole, Joseph M. Egan, Daniel H. Foil, Tyler N. Graf, Bryan D. Schindler, Glenn W. Kaatz, Nadja B. Cech

**Affiliations:** 1 Department of Chemistry/Biochemistry, The University of North Carolina Greensboro, Greensboro, North Carolina, United States of America; 2 Division of Infectious Disease, John Dingell Department of Veteran Affairs Medical Center and Department of Internal Medicine, Wayne State University School of Medicine, Detroit, Michigan, United States of America; University of Cambridge, UNITED KINGDOM

## Abstract

Bacterial efflux pumps are active transport proteins responsible for resistance to selected biocides and antibiotics. It has been shown that production of efflux pumps is up-regulated in a number of highly pathogenic bacteria, including methicillin resistant *Staphylococcus aureus*. Thus, the identification of new bacterial efflux pump inhibitors is a topic of great interest. Existing assays to evaluate efflux pump inhibitory activity rely on fluorescence by an efflux pump substrate. When employing these assays to evaluate efflux pump inhibitory activity of plant extracts and some purified compounds, we observed severe optical interference that gave rise to false negative results. To circumvent this problem, a new mass spectrometry-based method was developed for the quantitative measurement of bacterial efflux pump inhibition. The assay was employed to evaluate efflux pump inhibitory activity of a crude extract of the botanical *Hydrastis Canadensis*, and to compare the efflux pump inhibitory activity of several pure flavonoids. The flavonoid quercetin, which appeared to be completely inactive with a fluorescence-based method, showed an IC_50_ value of 75 μg/mL with the new method. The other flavonoids evaluated (apigenin, kaempferol, rhamnetin, luteolin, myricetin), were also active, with IC_50_ values ranging from 19 μg/mL to 75 μg/mL. The assay described herein could be useful in future screening efforts to identify efflux pump inhibitors, particularly in situations where optical interference precludes the application of methods that rely on fluorescence.

## Introduction

Bacterial efflux pumps are active transport proteins that function to extrude toxic compounds, including antimicrobial drugs, from the cell. These pumps serve to protect bacteria from damage by toxins, and can play a role in the development of resistance to antimicrobials [1–5]. For example, it has been shown that production of efflux pumps is up-regulated in drug resistant strains of many bacteria, including methicillin resistant *Staphylococcus aureus* [6–10]. Compounds that inhibit bacterial efflux pumps are of interest because of their potential to increase antimicrobial effectiveness [11]. Thus, our laboratory has been engaged in experiments to find new efflux pump inhibitors (EPIs) from natural product sources.

Current methods for evaluating efflux pump inhibitory activity rely on an efflux pump substrate that fluoresces only when it is located inside a cell (due to intercalation with DNA) [12]. The majority of existing protocols operate by pre-loading cells with the efflux pump substrate ethidium bromide, which gives them a high initial fluorescent intensity. The extent of efflux pump inhibition is then measured by comparing the rate of decrease in fluorescence intensity over time in the presence of varying amounts of the putative EPI [4,9,13–18]. Related experiments utilizing measurements based on the intracellular accumulation of fluorescent substrates have also been reported [9,19]. For accumulation experiments, fluorescence increases over time as the substrate diffuses into cells.

Ethidium bromide is attractive as an indicator of efflux pump inhibition because of extensive literature precedent and also because it has been established to be active via intracellular action, with literature precedent stretching back to the 1950s [12,20,21]. However, the existing methods for testing efflux pump inhibition with ethidium bromide gave false results in our study due to matrix quenching effects (the suppression of fluorescence by various components of the mixture) in crude extracts and even with some pure compounds. We endeavored to circumvent these quenching effects by developing a new mass spectrometry-based efflux pump inhibition assay. There is extensive literature support for the efflux pump inhibitory activity of flavonoids and related compounds [9–11,16,22–29]; thus, we sought to validate the new assay by comparing efflux pump inhibitory activity of a series of pure flavonoids. In addition, to test the validity of the new assay in a more crude sample matrix, we compared the efflux pump inhibitory activity of an extract from the botanical goldenseal (*Hydrastis canadensis*), which is known to contain EPIs [10,16], using both fluorescence and mass spectrometry-based approaches for data collection.

## Materials and Methods

### Preparation of plant material

The goldenseal leaf and petiole material used was cultivated in a woodland setting in Hendersonville, North Carolina, (N 35°24.2770', W 082°20.9930', 702.4 m elevation) and was made available by William Burch; this population has been utilized in previously published work [10,16] and is represented by a voucher (NCU583414) curated in the University of North Carolina at Chapel Hill herbarium. The goldenseal extract was prepared using previously described methods [10,30]. Dried plant material was macerated for at least 24 hr, and the methanol extract was subsequently separated from the plant material. This extract was dried in a rotary evaporator to reduce the volume of methanol, and partitioned against an equal volume of hexane. The resulting mixture was stirred for at least one hr and the layers were collected separately using a separatory funnel. The methanol partition was added to water and chloroform in a ratio of 1:5:4, stirred for at least 1 hr, then separated. The chloroform partition from this step was evaporated and used as the starting material for all experiments described herein, and will be referred to as “goldenseal extract” from this point forward.

### 96 well plate ethidium bromide accumulation assay

This assay is an adaptation of published ethidium bromide efflux-based assays [16–18] and previously published reports of measurements on intracellular accumulation of berberine and chloramphenicol [9,19]. All experiments presented here were performed in the 96 well plate format. Activity was tested using *Staphylococcus aureus* strain NCTC 8325–4 [31]. The final assay composition was 10% DMSO, 50% Muller-Hinton broth, 40% water (by volume), an estimated 1.6–1.8x10^8^ CFU/mL *S*. *aureus*, 1.25 μg/mL ethidium bromide, and a range of analyte concentrations. Data collection, and hence bacterial exposure to these conditions, was limited to 30 min. The alkaloid piperine was used as a positive control, as it is well established in the literature to be an EPI [25,32,33]. Each analyte concentration was tested in triplicate, and the positive control (a piperine dilution series ranging from 4.7 μg/mL to 300 μg/mL prepared via 2-fold dilution) was included on each plate. Fluorescence was measured using a BioTek SynergyH1 microplate reader (BioTek, Winooski, VT) with an excitation wavelength of 520 nm and emission wavelength of 600 nm at 1 min intervals for a total of 30 min. All experiments were performed in triplicate and error bars reported as standard deviation.

### Use of mass spectrometry to measure ethidium bromide accumulation

To enable mass spectrometric measurements of ethidium bromide accumulation, the experimental parameters were identical to those described in the previous section, except that the method of data collection was modified. The prepared samples were incubated at room temperature in a EMD Millipore MultiScreen fritted-bottom 96-well filter plate (pore size 0.22 μm, EMD Millipore, Darmstadt, Germany). At the conclusion of the 30 min incubation period, these were filtered simultaneously under vacuum into a receiving 96 well plate. All solutions were stored at 4°C prior to analysis.

Ethidium bromide in the bacterial supernatant was analyzed using high performance liquid chromatography (HPLC) electrospray ionization-mass spectrometry (ESI-MS). Liquid chromatography separations were achieved using a ThermoFinnigan Surveyer HPLC system (Thermo Finnigan, San Jose, CA). The autosampler was temperature controlled at 8°C, and the column (Agilent Prevail C_18_, 3 μm packing, 50 x 2.1 mm) was heated to 40°C. Sample injection volume was 5 μL and a flow rate of 0.2 mL/min was employed. Samples were eluted using binary gradients consisting of acetonitrile acidified with 1% acetic acid and deionized water (CH_3_CN:H_2_O) as follows:—0 min, 0:100; 1.5 min, 0:100; 2 min, 95:5; 10 min, 95:5; 10.5 min, 0:100; 18 min, 0:100. Mass spectrometry analyses were conducted with an LCQ DECA XP Plus ion trap mass spectrometer with electrospray ionization source (Thermo Fisher Scientific) using the following conditions: capillary temperature, 250°C; sheath gas flow, 10 (arbitrary units); no auxiliary gas; source voltage 4.5 kV; capillary voltage, 42 V; tube lens offset, -25 V. The instrument was operated in the positive ion mode with two scan events. The first was full scan, followed by the data-dependent CID fragmentation (50% collision energy) of *m/z* 314.20 (the [M]^+^ ion of ethidium). The selected ion chromatogram was plotted for the main product ion *m/z* 286, and its peak area was determined. All experiments were performed in triplicate and error bars set to standard deviation.

Mass spectrometry data were analyzed to determine an IC_50_ value for each test compound. The IC_50_ of piperine was defined as the midpoint between the peak area for vehicle control and that of the 300ppm piperine sample, similar to an approach employed previously [34]. Once determined for piperine, the same peak area was used as a set point for determining IC_50_ values of the test compounds on the same plate.

### Bacterial growth inhibition

MICs were determined according to Clinical Laboratory Standards Institute guidelines [35]. Solutions were prepared in 96 well plates with a final well volume of 250 μL, 2% DMSO in Mueller-Hinton broth, and variable concentrations of test compound or extract ranging from 4.7 to 150 μg/mL, prepared in triplicate. Duplicate plates of each experiment were employed, one inoculated with a bacterial concentration of 5x10^5^ CFU/mL, the other containing only analyte and vehicle. All plates were incubated for 18 hr at 37°C, after which turbidity at 600nm (OD_600_) was measured with a BioTek Synergy H1 microplate reader. To correct for background due to absorbance of the analyte compounds, the mean OD_600_ for each treatment without addition of bacteria was subtracted from the mean OD_600_ of treated wells. MIC was determined as the concentration where there was no statistically significant difference between the mean absorbance of the treated wells and that of the negative control (vehicle in broth).

## Results and Discussion

### Assay development and comparison of efflux pump inhibition assay methods

**Fig 1:**
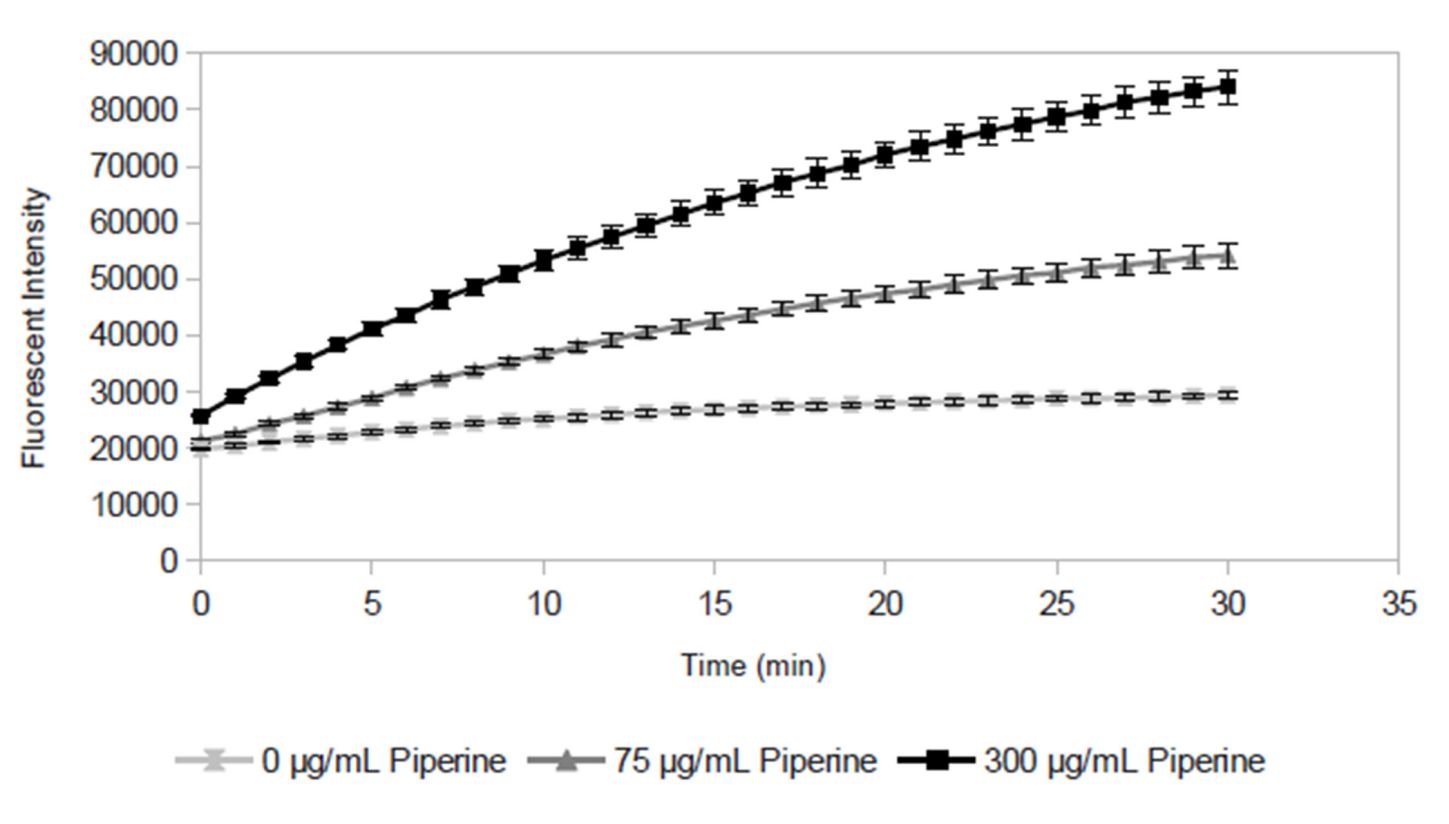
Change in absolute fluorescent intensity over time for *Staphylococcus aureus* exposed to ethidium bromide in the efflux pump inhibitor piperine. Fluorescence increases over time due to intracellular accumulation of ethidium bromide. The increase is more pronounced in the presence of piperine, which enhances intracellular accumulation of ethidium bromide by blocking efflux. Data points represent the mean of 3 samples, error bars represent standard deviation.

The first goal of our experiments was to determine the applicability of a fluorescence-based accumulation assay to measure the efflux pump inhibitory activity of various flavonoids. Towards this goal, we first validated the assay using a known efflux pump substrate (ethidium bromide) and a known EPI (piperine). As expected, when *S*. *aureus* is exposed to ethidium bromide, fluorescence increases over time (Fig 1). This increase is due to intracellular accumulation of ethidium, which fluoresces at 600 nm when it is intercalated with DNA [16]. Ethidium bromide is a substrate of NorA, a major chromosomally-encoded *Staphylococcus aureus* efflux pump [3,5]. Thus, the intracellular accumulation of ethidium bromide by *S*. *aureus* is counteracted by the action of NorA (and other efflux pumps). As evidence of this, the addition of piperine, a known NorA inhibitor, caused a more pronounced increase in fluorescence over time than was observed for the cells in the absence of the inhibitor (Fig 1).

**Fig 2:**
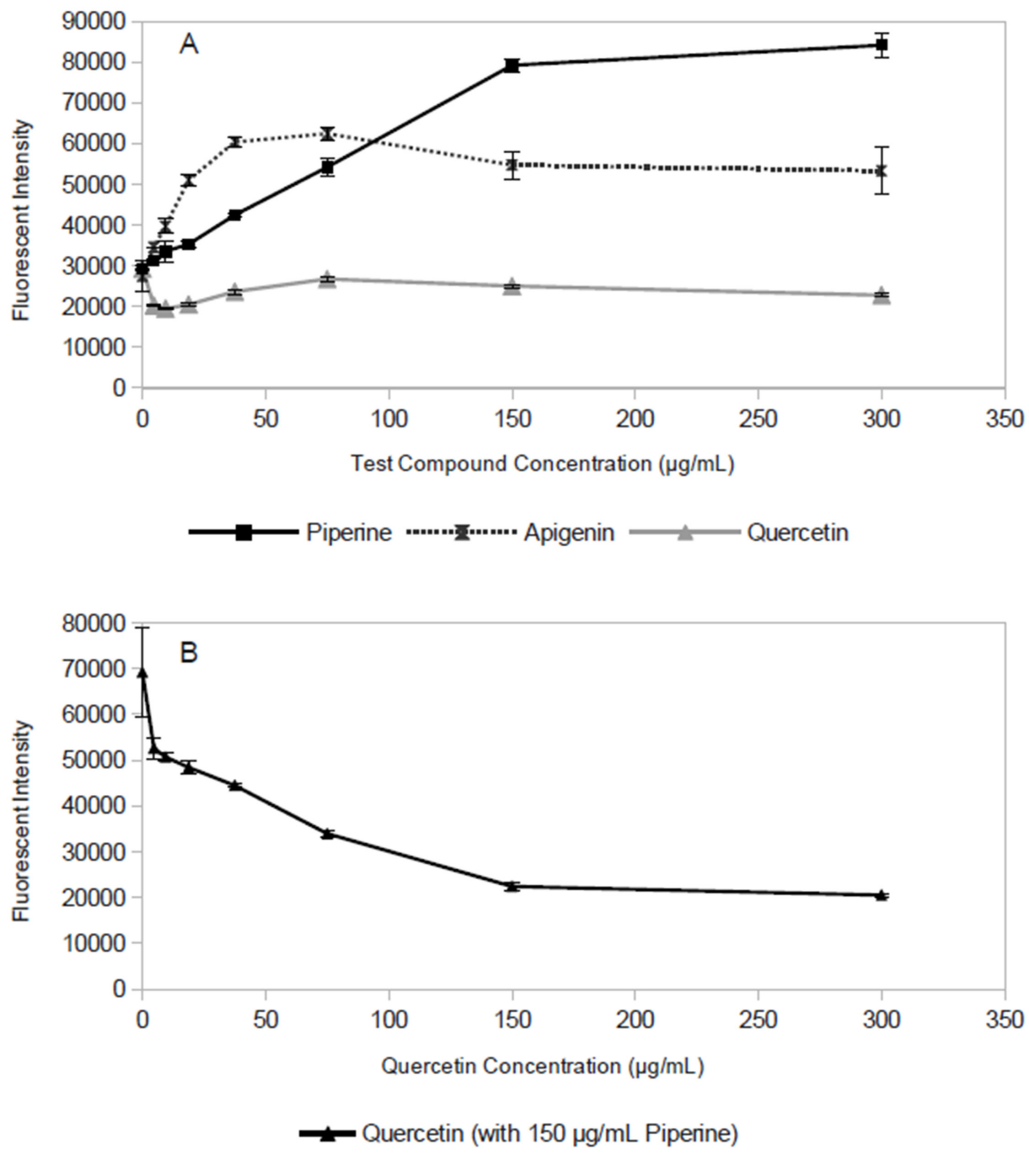
Change in fluorescence due to ethidium accumulation by *S*. *aureus* in the presence of putative inhibitors. (A) Dose-response curves for the flavonoids apigenin and quercetin. (B) Fluorescence observed for 150 μg/mL piperine in the presence of increasing concentrations of quercetin. Decrease in fluorescence with increasing quercetin concentration in (B) can be attributed to fluorescence quenching by the flavonoid. Data points represent the mean of triplicate measurements (biological replicates), error bars represent standard deviation.

Plotting the time-dependent accumulation assay data (Fig 1) at only a single time point (30 min) allows them to be represented as a dose-response curve (Fig 2A), with mean fluorescent intensity at one concentration on the y-axis, and test compound concentration on the x-axis. These curves demonstrate that ethidium bromide efflux is inhibited in the presence of piperine, and that this inhibition is dose-dependent (Fig 2A).

After the positive control was validated with the above methodology, the same approach was used to measure efflux inhibitory activity of several purified flavonoids. Flavonoids were chosen as test compounds because they are ubiquitous in plants, and by extension in plants that are used for food and for medicine [36,37]; and because numerous reports indicate that many flavonoids possess EPI activity [9–11,16,22–29]. Consistent with this precedent, the flavonoid apigenin displayed clear evidence of efflux pump inhibition (Fig 2A). However, the flavonoid quercetin appeared to be inactive. The unexpected negative result for quercetin led to the suspicion that optical matrix interference (quenching of ethidium bromide fluorescence) could interfere with accurate determinations of EPI activity of some compounds. To evaluate this possibility, a series of samples was prepared that included constant concentrations (150 μg/mL) of piperine, along with varying concentrations of the suspected quenching agent quercetin (Fig 2B). It is clear that as the concentration of quercetin increases in these solutions, the overall fluorescence of the mixture decreases, presumably due to quenching. Consistent with this observation, there are many reports of quenching in fluorescence-based assays, especially when plant extracts are involved [38–42].

**Fig 3:**
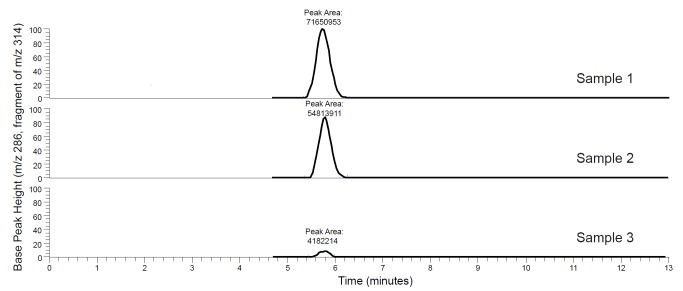
Representative selected chromatograms of filtered, spent broth showing a peak for the ethidium ion (MS-MS transition of *m/z* 314 to 286). Sample 1 is the negative control (*S*. *aureus* cultured for 30 min in Mueller Hinton broth with 1.25 μg/mL ethidium bromide and 10% DMSO), samples 2 and 3 were cultured under the same conditions as sample 1 with the addition of 75 μg/mL and 300 μg/mL piperine, respectively. All three peaks are normalized to a signal intensity of 3.45 x 10^6^. As piperine is added, efflux pumps are blocked, trapping the ethidium inside the cells and decreasing the quantity of ethidium (as indicated by the area of the ethidium peak) in the spent broth.

To circumvent the problem with optical matrix interference, a method was developed using liquid chromatography—mass spectrometry (LC-MS) to measure ethidium bromide concentrations in the spent broth filtrate. The concentration-response relationship for the data generated by the LC-MS method was expected to be the inverse of that generated by the measurement of fluorescence—as the concentration of an inhibitor increases, it traps the ethidium inside the bacterial cells, and the concentration of ethidium in the spent broth filtrate (as measured by LC-MS) should decrease. The known EPI piperine was again used for assay validation and it was observed that, as expected, the concentration of ethidium in the filtrate decreased with increasing concentration of piperine (Fig 3A). These results were plotted in a dose-response curve, with peak area displayed on the y-axis and concentration on the x-axis (Fig 3B).

With the LC-MS assay, it is possible to quantify the extracellular levels of ethidium *without relying on fluorescence*. Thus, if quercetin is in fact an active EPI and the negative results observed in the fluorescence based assay (Fig 2) are due to quenching, quercetin should have demonstrable activity in the new LC-MS based assay. Consistent with this expectation, the LC-MS based method showed quercetin to be active as an EPI (Fig 3B). Once the confounding effect of quenching is removed, it is apparent that quercetin is similar in its EPI activity to piperine, and apigenin is the most active EPI of the three. Furthermore, the LC-MS method showed a more typical dose-response relationship for apigenin (Fig 3B) compared to the fluorescence-based assay (Fig 2A), suggesting that apigenin quenches ethidium fluorescence at concentrations ≥ 38 μg/mL.

**Fig 4:**
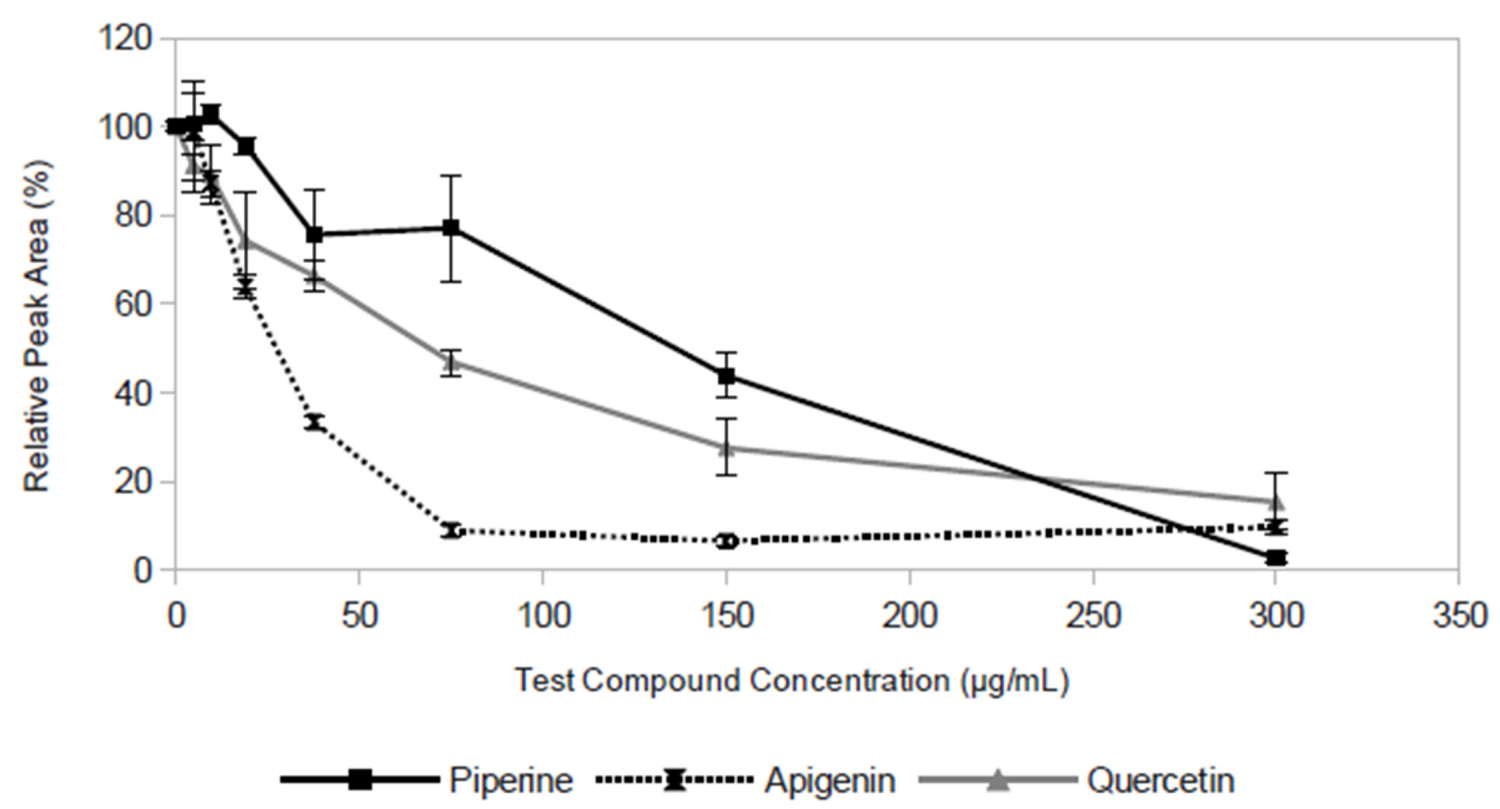
Efflux pump inhibitory activity of apigenin, piperine, and quercetin as indicated by LC-MS measurement of residual ethidium bromide in spent broth after a 30 min incubation. Relative peak area (expressed as a percentage) for ethidium is plotted as a function of concentration of the putative inhibitor. Data points represent the mean of 3 measurements (biological replicates), with error bars representing standard deviation.

To further evaluate the applicability of the LC-MS based efflux pump inhibition assay, we measured IC_50_ values (Table 1) for a series of six structurally diverse flavonoids and two known EPIs, the aforementioned compound piperine, and carbonyl cyanide m-chloro-phenylhydrazone (CCCP) [9,10,14,16,19]. All six flavonoids were active (sample data shown in Fig 4), with IC_50_ values ranging from 19 μg/mL (kaempferol and rhamnetin) to 75 μg/mL (quercetin, luteolin, and myricetin.) (Table 1). Piperine demonstrated an IC_50_ value in this assay between 75 μg/mL and 150 μg/mL, and CCCP was the most active of all compounds tested (IC_50_ 4.7 μg/mL, Table 1).

**Table 1:** Efflux pump inhibitory activity and antimicrobial activity of flavonoids.

Flavonoid	IC_50_ for efflux inhibition^a^	MIC^b^
Apigenin	38 μg/mL (140 μM)	>150 μg/mL (>560 μM)
Kaempferol	19 μg/mL (66.0 μM)	>150 μg/mL (>520 μM)
Rhamnetin	19 μg/mL (60.0 μM)	>150 μg/mL (>470 μM)
Quercetin	75 μg/mL (250 μM)	>150 μg/mL (>500 μM)
Luteolin	75 μg/mL (260 μM)	75 μg/mL (260 μM)
Myricetin	75 μg/mL (240 μM)	150 μg/mL (470 μM)
CCCP^c^	4.7 μg/mL (23 μM)	0.29 μg/mL (1.4 μM)

a: Efflux pump inhibition was measured via LC-MS analysis of ethidium in spent, filtered culture supernatant after a 30 min incubation in triplicate wells.

b: Growth inhibition was measured by optical density at 600nm (in triplicate) after an 18 hr incubation.

c: CCCP is an abbreviation for the compound carbonyl cyanide m-chloro-phenylhydrazone

**Fig 5:**
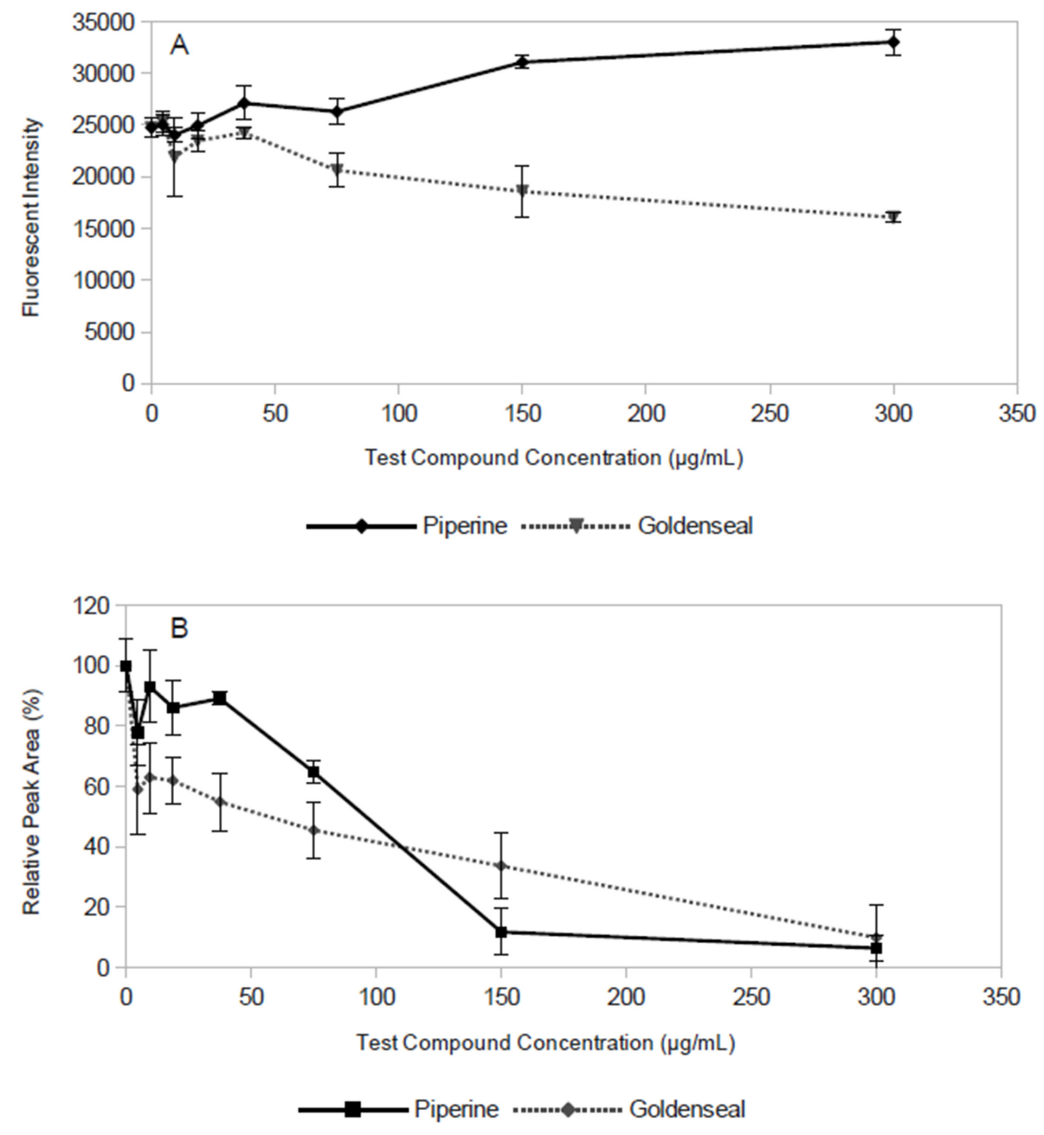
Efflux pump inhibition in *S*. *aureus* by a goldenseal (*Hydrastis canadensis*) extract. (A) Data collected using the fluorescence-based ethidium accumulation assay for a range of *H*. *canadensis* extract concentrations. (B) Data collected using the mass spectrometry-based ethidium accumulation assay. Incubation time was 30 min for both A and B, data represents mean of 3 samples, error bars represent standard deviation.

Finally, it was of interest to evaluate whether the mass spectrometry-based efflux assay would be applicable to samples with more complex matrices. Toward this goal, a botanical extract prepared from goldenseal (*Hydrastis canadensis*) was evaluated for EPI activity using both the fluorescence-based and mass spectrometry-based ethidium bromide accumulation assays. When assayed by the fluorescence-based method, the extract appeared inactive (no apparent IC_50_, Fig 5A), contradicting literature that indicates *H*. *canadensis* extracts contain EPIs [10,16]. However, when the activity of the extract was evaluated with the mass spectrometry-based assay, an IC_50_ value of 75 μg/mL was observed (Fig 5B). These results demonstrate that quenching can hamper the measurement of efflux pump inhibition for complex extracts using fluorescence, and that the interference can be overcome using the method developed herein. This finding is particularly important given that it may be useful in drug discovery efforts to screen complex extracts for the presence of EPIs.

### Potential interference by growth effects

Many botanical compounds possess antimicrobial activity. Thus, it was important to evaluate whether growth effects might confound the measurements of efflux pump inhibition. Towards this goal, the flavonoids, controls, and the goldenseal extract were screened for inhibition of bacterial growth across a range of concentrations, from 4.7 μg/mL to 150 μg/mL. Only three samples, the flavonoids luteolin and myricetin, and the known EPI CCCP, demonstrated measureable MICs under these conditions (Table 1). To determine whether growth inhibition by these compounds was likely to confound data interpretation, the two inhibitory flavonoids (luteolin and myricetin) were incubated with test strains under the experimental conditions at twice their IC_50_ values (Table 1). *S*. *aureus* cells were tested for loss of viability in the presence of these flavonoids (as well as in the presence of the flavonoid apigenin which does not inhibit bacterial growth) by replicating the experimental conditions of bacterial, ethidium bromide, broth and DMSO content, and plating aliquots of the resulting culture onto supplemented Mueller-Hinton agar at 0, 15 and 30 min time points. No loss of viability was observed after a 30 min exposure to the experimental conditions, as determined by colony count enumeration (S1 Fig). Additionally, to further evaluate the potential for simple toxicity to confound evaluation of data in this assay, the commercial antibiotics gentamicin and nafcillin were subjected to the mass spectrometry-based efflux pump inhibition assays, and no IC_50_ was observed to the maximum concentration tested of 100 μg/mL (S2 Fig). The highest tested concentration for the antibiotics was well above their reported MICs against *Staphylococcus aureus* (~0.5 μg/mL [43,44]). Collectively, these results suggest that growth inhibition does not confound the measurements of efflux pump inhibitory activity reported in Table 1.

## Conclusions

In light of the health risks posed by the increased occurrence of antibiotic resistant bacterial strains, the need for reliable methods for their study is of high importance. The mass spectrometry-based method to quantitatively investigate efflux pump inhibition is just such a tool, and as such is expected to be of high value to the scientific community. Our study shows that misleading results (false negative in the case of assays that rely on ethidium bromide accumulation) can be obtained when screening crude extracts and even pure compounds with fluorescence-based efflux pump inhibition assays. The new method presented here circumvents these problems. Additionally, the ubiquity of activity in the flavonoids tested in the validation process of this assay reinforces the importance of this class of natural products in the reversal of efflux-pump mediated drug resistance.

## Supporting Information

S1 Fig Time series data evaluating the viability of *Staphylococcus aureus* cultures exposed to experimental conditions. Conditions are as follows: 10% DMSO, 50% Muller-Hinton broth, 40% water (by volume), with 1.25 μg/mL ethidium bromide, for a maximum of 30 min. Test compounds include two flavonoids that inhibit the growth of this strain (luteolin and myricetin), and one that does not (apigenin) (Table 1). (EPS)
(TIF)

S2 Fig Efflux pump inhibitory data generated via the mass spectrometry-based assay performed on the known antibiotic compounds gentamicin and nafcillin. Also shown is a control dose-response curve performed on the positive control piperine.
(TIF)
