# Supplementary figures and images for: A Mass Spectrometry-Based Assay for Improved Quantitative Measurements of Efflux Pump Inhibition
Source: PLoS One. 2015 May 11;10(5):e0124814. doi: 10.1371/journal.pone.0124814 (PMC4427306; doi:10.1371/journal.pone.0124814)

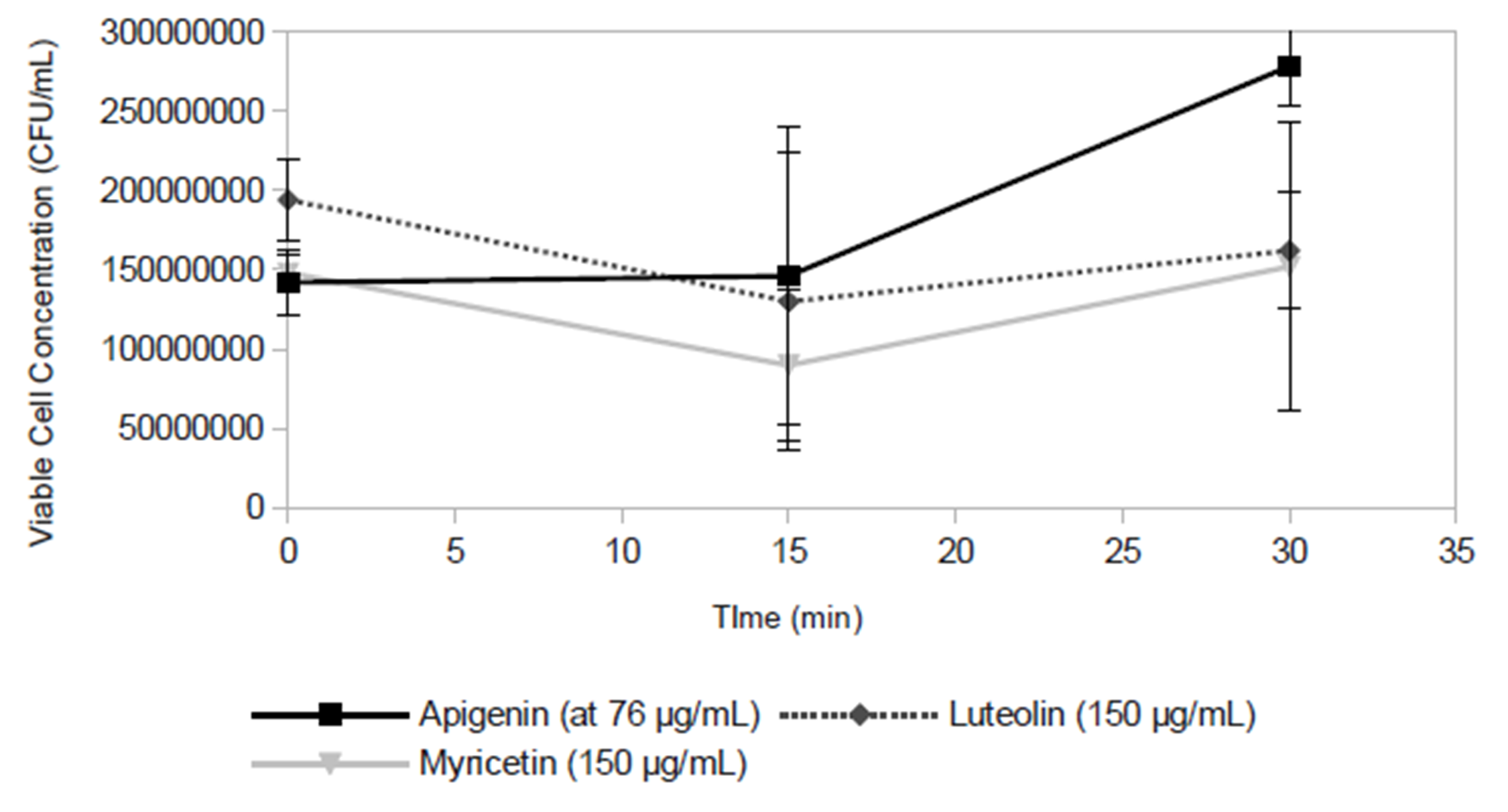

Supplement: S1 Fig — Conditions are as follows: 10% DMSO, 50% Muller-Hinton broth, 40% water (by volume), with 1.25 μg/mL ethidium bromide, for a maximum of 30 min. Test compounds include two flavonoids that inhibit the growth of this strain (luteolin and myricetin), and one that does not (apigenin) (Table 1). (EPS) (TIF) [file pone.0124814.s001.tif]

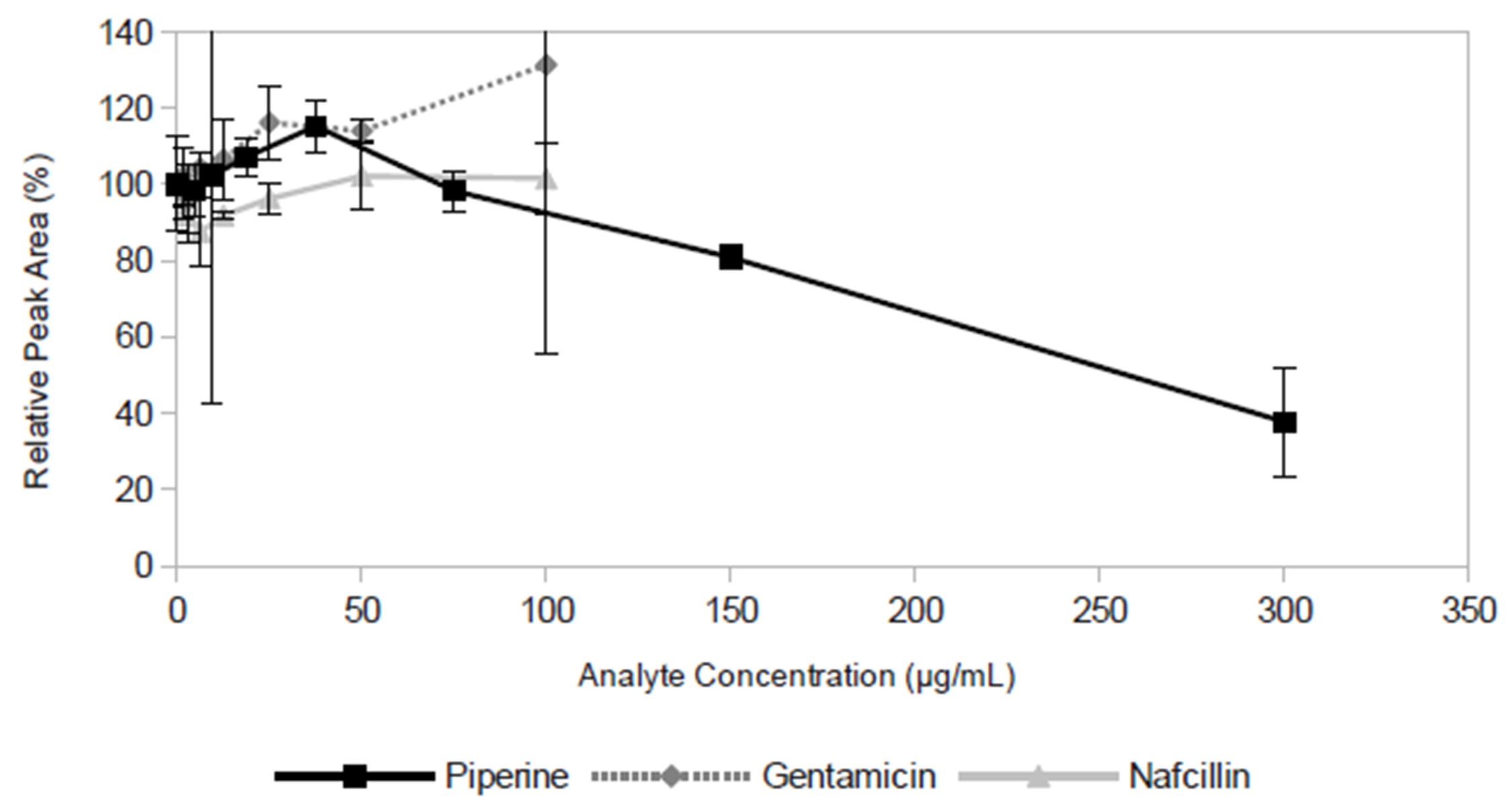

Supplement: S2 Fig — Also shown is a control dose-response curve performed on the positive control piperine. (TIF) [file pone.0124814.s002.tif]
